# Robotic distal pancreatectomy using a novel surgical robot platform “hinotori™” (with video)

**DOI:** 10.1002/jhbp.12137

**Published:** 2025-03-12

**Authors:** Takao Ide, Noriyuki Egawa, Kotaro Ito, Tomokazu Tanaka, Hirokazu Noshiro

**Affiliations:** ^1^ Department of Surgery Saga University Faculty of Medicine Saga Japan

## Abstract

With accompanying video, Ide and colleagues report their first clinical experience using the new Japanese surgical robot hinotori™ for robotic distal pancreatectomy, with favorable short‐term surgical results. Their findings suggest that the hinotori™ surgical robot provides a feasible and reproducible surgical procedure for safe and effective robotic distal pancreatectomy.

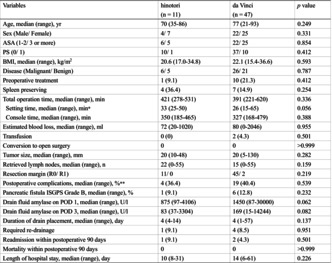

In the field of hepatobiliary‐pancreatic surgery, the da Vinci™ Surgical System (Intuitive Surgical Inc., CA) has been the only leader‐follower surgical robot.^1^ While new surgical robot platforms for clinical practice have recently become available,^2^ no reports have focused on comparing surgical results with the da Vinci™ Surgical System in pancreatectomy. We present our first clinical experience in robotic distal pancreatectomy utilizing a new surgical robot, “hinotori™” (Medicaroid Corporation, Kobe, Japan).

The surgical technique involved placing five trocars in the upper abdomen, including one for an assistant (Figure S1a). After placing the patient in the supine position with 10° head up and 5° left side up, the operation unit was rolled from the right side (Figure S1b). The pivot was set using a pivot pointer on each trocar of the robotic arms (Figure S1c). The cockpit surgeon performed all procedures, excluding port placement, using a vessel sealing device and pancreatic transection (Video S1).

Fifty‐eight consecutive patients underwent robotic distal pancreatectomy (hinotori: *n* = 11; da Vinci: *n* = 47) between March 2013 and November 2024 (Table 1). Postoperative pancreatic fistula was observed in 9.1% and 12.8% of the cases in the hinotori and da Vinci groups, respectively. Drain fluid amylase levels on postoperative days 1 and 3 were lower in the hinotori group. The oncological performance was comparable between the hinotori and da Vinci groups.

**TABLE 1:** Patient characteristics and short‐term surgical results.

Variables	hinotori (*n* = 11)	da Vinci (*n* = 47)	*p* Value
Age, median (range), year	70 (35–86)	77 (21–93)	.249
Sex (male/female)	4/7	22/25	.331
ASA (1–2/3 or more)	6/5	22/25	.854
PS (0/1)	10/1	37/10	.412
BMI, median (range), kg/m^2^	20.6 (17.0–34.8)	22.1 (15.4–36.6)	.593
Disease (malignant/benign)	6/5	26/21	.787
Preoperative treatment	1 (9.1)	10 (21.3)	.412
Spleen preserving	4 (36.4)	7 (14.9)	.254
Total operation time, median (range), min	421 (278–531)	391 (221–620)	.336
Setting time, median (range), min^a^	33 (25–50)	26 (15–65)	.056
Console time, median (range), min	350 (185–465)	327 (168–479)	.388
Estimated blood loss, median (range), mL	72 (20–1020)	80 (0–2046)	.955
Transfusion	0 (0)	2 (4.3)	.501
Conversion to open surgery	0	0	>.999
Tumor size, median (range), mm	20 (10–48)	20 (5–130)	.282
Retrieved lymph nodes, median (range), *n*	22 (0–55)	15 (0–55)	.159
Resection margin (R0/R1)	11/0	45/2	.219
Postoperative complications, median (range), %^b^	4 (36.4)	19 (40.4)	.539
Pancreatic fistula ISGPS Grade B, median (range), %	1 (9.1)	6 (12.8)	.232
Drain fluid amylase on POD 1, median (range), U/L	875 (97–4106)	1450 (87–30 000)	.062
Drain fluid amylase on POD 3, median (range), U/L	83 (37–3304)	169 (15–14 244)	.082
Duration of drain placement, median (range), day	4 (4–14)	4 (1–57)	.137
Required re‐drainage	1 (9.1)	4 (8.5)	.951
Readmission within postoperative 90 days	1 (9.1)	2 (4.3)	.501
Mortality within postoperative 90 days	0	0	>.999
Length of hospital stay, median (range), day	10 (8–31)	14 (6–61)	.226

*Note*: Data are presented as number (%) unless specified.

Abbreviations: ASA, American Society of Anesthesiologists Physical Status; BMI, body mass index; ISGPS, International Study Group of Pancreatic Surgery; POD, postoperative days; PS, performance status.

^a^ Duration from skin incision to starting work in the console (cockpit).

^b^ Defined as grade II or higher according to the Clavien–Dindo classification.

The advantages of the hinotori system over the da Vinci system are as follows: (1) each arm has eight axes, which brings more flexibility and reduces the interference between the arms; (2) it employs a “docking‐free” design using a software program, which provides a sufficient working space around the trocars^3^ (Figure S1d); (3) it is adaptable to any trocar and hopping between trocars is easy, leading to easier stapling for pancreatic transection; (4) it has a lower price, with a comparable maintenance fee^2^; and (5) it has been reported to be a possible platform for remote surgery.^2^ Meanwhile, the disadvantages are as follows: (1) it requires a longer setting time; (2) the number of available instrument types is low; (3) the range of joint motion in the instrument is smaller; (4) it tends to lose sight of the instruments on the screen; and (5) the collision alarm is sensitive, which sometimes forces operations to be stopped automatically. Hence, understanding the features of each robotic system is important for establishing a surgical strategy for patients with various conditions.

This first clinical series of robotic pancreatectomies using the hinotori system showed favorable short‐term surgical results. The hinotori surgical robot provides a feasible and reproducible surgical procedure for the secure performance of robotic distal pancreatectomy.

## CONFLICT OF INTEREST STATEMENT

The authors declare no conflicts of interest in association with the present study.

## Supporting information


Figure S1.

Video S1.
