# Supplementary figures and images for: Robotic distal pancreatectomy using a novel surgical robot platform “hinotori™” (with video)
Source: J Hepatobiliary Pancreat Sci. 2025 Mar 12;32(6):415–7. doi: 10.1002/jhbp.12137 (PMC12188151; doi:10.1002/jhbp.12137)

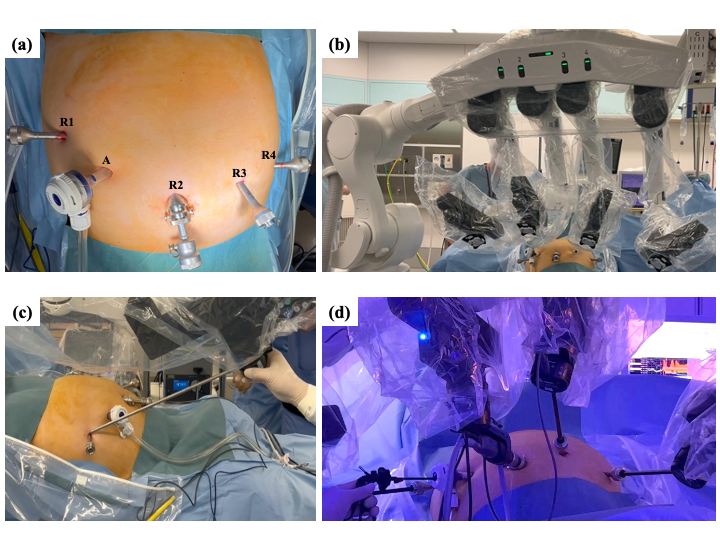

Supplement: Supplementary file 1 — Figure S1. [file JHBP-32-415-s002.tiff]
